# Coronavirus Disease 2019 Pandemic: Health System and Community Response to a Text Message (Text4Hope) Program Supporting Mental Health in Alberta

**DOI:** 10.1017/dmp.2020.114

**Published:** 2020-04-22

**Authors:** Vincent I. O. Agyapong

**Affiliations:** Department of Psychiatry, Faculty of Medicine and Dentistry, University of Alberta, Edmonton, Alberta, Canada

**Keywords:** Alberta, COVID19, support, Text4Hope, text messages

## Abstract

In an effort to support the mental health of Albertans during the coronavirus disease 2019 (COVID-19) pandemic, Alberta Health Services launched a supportive text message (Text4Mood) program on March 23, 2020. The program was simultaneously approved for funding by the 6 regional health foundations and launched within 1 week of conception. Residents of Alberta can subscribe to the program by texting “COVID19HOPE” to a sort code number. Each subscriber receives free daily supportive text messages, for 3 months, crafted by a team of clinical psychologists, psychiatrists, mental health therapist, and mental health service users. Within 1 week of the launch of Text4Hope, 32 805 subscribers had signed up to the program, and there have been expressions of interests from other jurisdictions to implement a similar program to support the mental health of those in quarantine, isolation, or lockdown.

The severe acute respiratory syndrome coronavirus 2 (SARS-CoV-2) outbreak started in the Hubei province of China in December 2019 and was declared a global pandemic by the World Health Organization (WHO) on March 11, 2020.^1^ As of April 8, 2020, SARS-CoV-2 had spread to 212 countries and territories around the world, with 1 356 780 confirmed cases of coronavirus disease 2019 (COVID-19) and 79 385 deaths.^2^ In Canada, there were 19 289 confirmed COVID-19 cases on April 8, 2020, of which 1423 were in the province of Alberta.^3^ A recent rapid review of 24 published studies on pandemics reported negative psychological effects of quarantine, including posttraumatic stress symptoms, confusion, and anger. Stressors included longer quarantine duration, infection fears, frustration, boredom, inadequate supplies, inadequate information, financial loss, and stigma about the infection.^4^ Universally, there exist wide treatment gaps for mental disorders,^5^ and these gaps tend to widen during humanitarian emergencies due to disruption to health systems.^6^ A public health systems approach, therefore, is needed to address the mental health challenges associated with pandemics and other natural disasters.^6^ In recent years, there has been growing interest in the use of supportive text messaging to address the mental health treatment gap at the population level.^7^ In 2 randomized controlled trials, patients with depression showed symptom reduction on standardized self-report compared to a similar patient group not receiving messages (with large effect sizes: Cohen’s d = 0.85^21^, Cohen’s d = 0.67^22^).^8,9^ In 2 user satisfaction surveys, over 80% of subscribers to similar programs reported that the programs improved their mental health.^10,11^ In another randomized controlled trial to evaluate the effectiveness of an addiction-related supportive text messaging service to improve treatment outcomes for patients with alcohol use disorder, the intervention group’s mean first day to drink was over twice the length of the control group (eg, approximately 60 vs 26 days, respectively, with a mean difference of 34.97 and 95% CI of -5.87 to 75.81).^12^

Based on the emerging evidence of feasibility and in an effort to help close the COVID-19 pandemic-induced psychological treatment gap for all residents of Alberta, on Monday, March 23, 2020, Alberta’s Chief Medical Officer launched a free supportive text message service, Text4Hope, on behalf of Alberta Health Services. Text4Hope allows subscribers to receive 3 months of daily supportive text messages with or without weblinks to online mental health resources. Individuals can subscribe to Text4Hope by simply texting “COVID19HOPE” to a short code number. The messages were crafted by a team of clinical psychologists, psychiatrists, mental health therapists, and mental health patients to address stress, anxiety, and depression. An example of the messages sent is: “When bad things happen that we can’t control, we often focus on the things we can’t change. Focus on what you can control; what you can do to help yourself (or someone else) today.” The Text4Hope program was modeled after the Text4Mood program, which was launched in Northern Alberta in 2016 and played a vital role in supporting the mental health of residents of Fort McMurray during the wildfires of the same year. Text4Mood was subsequently recognized as a mental health innovation^13^ by the Mental Health Innovations Network, which is headquartered at the Department of Mental Health and Substance Abuse of the WHO.

The Text4Hope program was developed within 48 hours of funding confirmation by a team of academics and clinicians based at the Department of Psychiatry, University of Alberta, and Alberta Health Services. The program is currently receiving sponsorship from 6 health foundations in Alberta, namely, the Mental Health Foundation, Calgary Health Trust, the University Hospital Foundation, the Alberta Children’s Hospital Foundation, the Royal Alexandra Hospital Foundation, and the Alberta Cancer Foundation with support from the University of Alberta. Remarkably, all of the health foundations responded within 24 hours to the Text4Hope funding support call that was put out by Alberta Health Services Foundation Relations, with over a million dollars committed to the program. One week after the launch, the program received 32 805 subscriptions, and the subscriber list keeps growing by the minute each day. Subscribers are invited to voluntarily complete a baseline questionnaire that includes their demographic information, as well as standardized measures for stress, anxiety, and depression. Survey links will also be sent to each subscriber at 6 and 12 weeks to assess program benefits and subscriber satisfaction. This study has ethical approval from the Research and Ethics Board of the University of Alberta (Pro00086163), and a full research protocol would be published in due course. Since the launch of the program, there have been interests in launching similar programs in other jurisdictions in Canada, Australia, and Ghana. The massive subscription to the supportive text messaging program in Alberta suggests that such a service is feasible and acceptable to end-users and can potentially support the mental health of people in self-isolation, quarantine, or lockdown during pandemics and other emergencies in Canada and around the world.
